# LipidFun: a database of lipid functions

**DOI:** 10.1093/bioinformatics/btaf110

**Published:** 2025-03-13

**Authors:** Wen-Jen Lin, Chia-Hsin Liu, Ming-Siang Huang, Pei-Chun Shen, Hsiu-Cheng Liu, Meng-Hsin Tsai, Yo-Liang Lai, Yu-De Wang, Mien-Chie Hung, Nai-Wen Chang, Wei-Chung Cheng

**Affiliations:** School of Medicine, China Medical University, Taichung 404333, Taiwan; Graduate Institute of Biomedical Science, China Medical University, Taichung 404333, Taiwan; Cancer Biology and Precision Therapeutics Center, China Medical University, Taichung 406040, Taiwan; Department of Computer Science & Information Engineering, Asia University, Taichung 41354, Taiwan; Cancer Biology and Precision Therapeutics Center, China Medical University, Taichung 406040, Taiwan; Cancer Biology and Precision Therapeutics Center, China Medical University, Taichung 406040, Taiwan; Cancer Biology and Precision Therapeutics Center, China Medical University, Taichung 406040, Taiwan; Department of Radiation Oncology, China Medical University, Taichung 404328, Taiwan; Graduate Institute of Biomedical Science, China Medical University, Taichung 404333, Taiwan; Department of Urology, China Medical University, Taichung 404333, Taiwan; Cancer Biology and Precision Therapeutics Center, China Medical University, Taichung 406040, Taiwan; Institute of Biochemistry and Molecular Biology, China Medical University, Taichung 404328, Taiwan; Molecular Medicine Center, China Medical University Hospital, China Medical University, Taichung 404328, Taiwan; Department of Biotechnology, Asia University, Taichung 413305, Taiwan; Department of Medical Research, National Taiwan University Hospital, Taipei 100229, Taiwan; Graduate Institute of Biomedical Science, China Medical University, Taichung 404333, Taiwan; Cancer Biology and Precision Therapeutics Center, China Medical University, Taichung 406040, Taiwan; The Ph.D. Program for Cancer Biology and Drug Discovery, China Medical University and Academia Sinica, Taichung 404328, Taiwan

## Abstract

**Motivation:**

Lipids play crucial roles in various biological functions and diseases. However, a gap exists in databases providing information of lipids functions based on curated information. Consequently, LipidFun is purposed as the first lipid function database with sentence-level evidence detailing lipid-related phenotypes and biological functions.

**Results:**

Potential lipid functions were extracted from the biomedical literature using natural language processing techniques, with accuracy and reliability ensured through manual curation by four domain experts. LipidFun constructs classification systems for lipids, biological functions, and phenotypes for named entity recognition. Sentence-level evidence is extracted to highlight connections to lipid-associated biological processes and diseases. Integrating these classification systems and a large amount of sentence-level evidence allows LipidFun to provide an overview of lipid–phenotype and lipid–biological function associations through concise visualizations. Overall, LipidFun unravels the relationships between lipids and biological mechanisms, underscoring their overarching influence on physiological processes.

**Availability and implementation:**

LipidFun is available at https://lipidfun.bioinfomics.org/.

## 1 Introduction

A diverse class of biomolecules, lipids play essential roles in various biological processes and diseases. Lipids are fundamental components of cell membranes, helping to maintain membrane integrity and fluidity and modulating cellular signaling pathways (Sezgin *et al.* 2017). Moreover, lipids serve as an energy reservoir (Olzmann and Carvalho 2019), inducers of apoptosis (Grösch *et al.* 2012) or ferroptosis (Kagan *et al.* 2017), and precursors for synthesizing vital molecules such as hormones, vitamins, and eicosanoids (Chiurchiù *et al.* 2018). Several diseases have also been closely associated with aberrant lipid metabolism, including cardiovascular diseases (Mach *et al.* 2020), metabolic disorders (Unger *et al.* 2010), neurodegenerative disease (Yadav and Tiwari 2014), and cancers (Corn *et al.* 2020). Despite their significance in biological functions and diseases, limited databases currently provide comprehensive information on lipids to substantiate their roles, similar to how the gene ontology (The Gene Ontology Consortium 2019) and DisGeNet (Piñero *et al.* 2020) databases contribute to gene research.

With advances in lipidomics, researchers can now precisely and efficiently profile hundreds to thousands of lipid species (Wang *et al.* 2016, Ni *et al.* 2023). Over the past two decades, numerous studies have reported associations between various biological conditions and phenotypes with specific lipid species or lipid classes, which are comprised of unique subgroups of lipids sharing structural similarities (Zou *et al.* 2020, Bae *et al.* 2022, Liu *et al.* 2024). For example, arachidonic acid and prostaglandin E2 play critical roles in inflammation (Calder 2006). Conversely, ceramides (Cers) represent a group of lipids that promote apoptosis, and the accumulation of various triacylglycerols (TAGs) has been associated with obesity (Lafontan and Langin 2009), nonalcoholic fatty liver disease (Kawano and Cohen 2013), and cardiovascular diseases (Nordestgaard and Varbo 2014). Therefore, integrating this information to annotate lipid functions could significantly advance the lipid biology field. Nonetheless, this task remains challenging, and only a few databases currently address this issue.

The LipidPedia (Kuo and Tseng 2018) and LipiDisease (More *et al.* 2021) databases link lipids with diseases or biomedical information, systematically and precisely exploring the functions of diverse lipids and providing evidence continues to present challenges. However, some remaining issues still require to be overcome. Firstly, current databases only provide evidence at the document level, where lipid and function terms co-occur within a single article without relationship identification or curation. To our knowledge, no lipid database contains sentence-level evidence of lipid functions, which describes the lipid-function association in a single sentence and is essential for researchers to confirm its correctness. Secondly, the article search should focus not only on specific lipid species but also on lipid classes, as the latter also play an equally crucial role in exerting biological functions. Neglecting lipids documented at the class level could substantially reduce available articles and severely impact common categories such as phosphatidylcholine (PC), TAG, or Cer since the lipids in these categories often share common metabolic pathways and enzymes and are investigated as a unified class (Gillespie *et al.* 2022, Kanehisa *et al.* 2022). Thirdly, classification systems for lipids or functions are needed for the database to gather the evidence of downstream entities for the upstream ones. For example, if one PC species is related to inflammation, it can be implied that the PC class is also related to inflammation. If a user wants to examine the PC-inflammation association, the database can easily extract the evidence for the PC class and its species. Finally, there is no systematic network analysis to illustrate the interrelationships between affected lipids or functionalities and shed light on their collective impact in various biological contexts.

To address these challenges, we present LipidFun, the first database containing information systematically retrieved and curated from the lipid-related literature to explore lipid functions based on both lipid species and classes. Moreover, LipidFun incorporates the classification systems of biological function and phenotypes based on medical subject headings (MeSH) and an expert-curated lipid classification system created by integrating the LIPID MAPS database(Conroy *et al.* 2023). This approach ensures the generation of a well-structured and hierarchically organized lipid network, enhancing the accuracy and consistency of lipid classification and functional characterization. We also leverage natural language processing (NLP) techniques to extract lipid-associated information from the scientific literature, including biological functions and phenotypes, enhancing LipidFun’s comprehensiveness. By incorporating sentence-level evidence with lipid-oriented relation extraction, LipidFun efficiently retrieves lipid information from a vast body of scientific literature, providing a valuable resource for further manual curation. These comprehensive features empower LipidFun to deliver precise and reliable insights into the functional roles of lipids to the greatest extent, facilitating advancements in lipidomics research and furthering our understanding of their crucial contributions to various biological processes.

## 2 Materials and methods

### 2.1 Definition and classification of lipids

Our methodology for delineating lipid definition and classification is grounded on the framework provided by the LMSD. This expansive repository contains diverse biologically pertinent lipid structures with detailed annotations. We used two main strategies, MeSH IDs and a keyword-driven search, to annotate lipid terms in the literature. Firstly, our standardization process involved mapping lipids from the LMSD to corresponding MeSH IDs using the PubChem database, followed by a further collection of MeSH IDs under the lipids category (Tree number: D10) in the MeSH database to broaden our controlled vocabulary. However, while focusing on lipid species, this method may inadequately represent common lipid classes, such as lysophosphatidic acid, potentially omitting a significant volume of articles reporting lipid terms at the class level, which are known to be associated with numerous diseases and biological functions. In order to address this gap, our second step involved extracting keywords corresponding to lipid categories from the LMSD to enrich our search lexicon. Here, the lipid category names were specifically collected, excluding categories without informational content, such as “other glycerolipids” or “other glycerophospholipids.”

Furthermore, the names composed of multiple components, such as “N-acylsphingosines (ceramides)” or “lysosphingomyelins and lysoglycosphingolipids” were disassembled into their component elements, “N-acylsphingosines” with “ceramides” and “lysosphingomyelins” with “lysoglycosphingolipids,” respectively. LMSD IDs ending with “0000,” where the skeleton categories were excluded, belong to the critical lipid classes in metabolic pathways, such as PC and phosphatidylethanolamine. For these classes, we collated the corresponding Kyoto Encyclopedia of Genes and Genomes, Chemical Entities of Biological Interest, and PubChem IDs from the LMSD and expanded our collection of synonyms through these databases. This augmentation increased the yield of pertinent sentences in our ultimate keyword search by over 20%.

Furthermore, using the LMSD classification framework enabled the categorization of lipids within upstream and downstream pathways. One note of caution is that since MeSH is not exclusively a lipid-centric database, its classifications can diverge from the LMSD. For example, MeSH classifies sphingomyelins under phospholipids, while they are categorized under sphingolipids in the LMSD. We defaulted to the LMSD standards for reclassification when we encountered such discrepancies. Additionally, some categories only documented in MeSH, such as lysophospholipids, were incorporated into our classification system to ensure that all lipids, whether identified by MeSH ID or keyword search, were accurately classified and represented in the classification network.

### 2.2 Definition and classification of phenotypes and biological functions

We integrated data from four key databases: Pubtator, MeSH, HPO, and UMLS, to create a comprehensive phenotype dictionary containing approximately 20 000 terms associated with various phenotypes and diseases. The integration of these databases enhanced the breadth of the phenotype dictionary, surpassing the coverage provided by any individual database (see Supplementary Fig. S1). This phenotype dictionary was subsequently utilized to annotate phenotype names in sentences using a pattern matching approach. We also meticulously curated the biological function dictionary by harmonizing terms in the UMLS T070 branch and filtered from the biological process and molecular function gene ontology categories. These were then integrated with node information derived from specific branches of the MeSH database (G03, G04, G06, G12, G02.111, G05.113, G05.135, G05.297, G05.308, and G05.558), ensuring a robust and comprehensive compilation of biological function terms. During preprocessing, the abstracts were tokenized, sentence-segmented, and standardized to lowercase, enabling meticulous string matching with the phenotype or biological function dictionary, completing the annotation phase. This string matching used a hybrid approach, combining pattern-based methods, and regular expressions. Here, terms aligned with MeSH IDs were diligently recorded, while those lacking these identifiers were excluded.

### 2.3 Sentence-level evidence

We initially collected MeSH IDs and vocabularies related to lipids, phenotypes, and biological functions to annotate approximately 27 million abstracts in the PubTator 2 database. These abstracts were tokenized and sentence-segmented, and those containing both a lipid and a phenotype or biological function were extracted for further analysis. Our co-occurrence method preliminarily identified 591 577 and 552 238 abstracts for lipid-associated biological functions and phenotypes, respectively, and the large language model (GPT-3.5-turbo-0301 model) was used to assist in extracting meaningful associations. To evaluate the reliability of associations, we randomly selected sentences based on Lipid–Biological Function and Lipid–Phenotype pairs, allowing for up to three instances for each pair. This approach enables a comprehensive assessment of all potential associations during the manual curation process conducted by four domain experts. Our selection process was randomized across the entire dataset, without restricting it to any specific subset of abstracts. As of now, a total of 7548 sentences have been assessed in LipidFun, comprising 3453 sentences related to lipid–biological functions (Supplementary Table S1) and 4095 sentences pertaining to lipid–phenotypes, drawn from 6463 abstracts (Supplementary Table S2), as described in Supplementary Methods. Currently, the model had a PPV of 0.90 and 0.91 for lipid–biological function and lipid–phenotype, respectively (Supplementary Tables S1 and S2) in predicting both lipid–biological function and lipid–phenotype associations. Finally, LipidFun includes 3011 sentences related to lipid-associated biological functions and 3608 sentences related to phenotypes, both confirmed through human curation (Table 1).

**Table 1.** Summary of LipidFun.^a^

Category	Type	Number of records
Named entity	Lipids	2490 lipids
	Biological function	484 functions
	Phenotype	2940 phenotypes
Article	Lipid–biological function	314 574 papers
	Lipid–phenotype	280 376 papers
Sentence	Lipid–biological function	591 577 (3011^a^) sentences
	Lipid–phenotype	552 238 (3608^a^) sentences

a The parenthetical number indicates the number of manually curated sentences.

### 2.4 Definition of association index

Some lipids are linked to multiple phenotypes or biological functions, while others are connected to a limited set or just one. To quantify such multifaceted property of various lipids in terms of phenotypes or biological functions association, we designed two types of association indexes, DSI and DPI, for specificity and pleiotropy, respectively. Theoretically ranging from 0 to 1, the DSI assesses this characteristic of lipids, indicating whether a lipid is linked to many (low DSI) or a few (high DSI) phenotypes or biological functions. The DSI is calculated as follows:


(1) $$\mathrm{DSI}=\log_{2}(N_{d}/N_{T})/\log_{2}(1/N_{T})\;$$

where *N_d_* is the number of phenotypes or biological functions associated with the lipid, and *N_T_* is the total number of phenotypes or biological functions in LipidFun.

Theoretically ranging from 0 to 1, the DPI is comparable to the DSI but assesses whether multiple phenotypes or biological functions associated with lipids share a common category under the MeSH phenotype (low DPI) and biological processes classification or if they are distributed across different categories (high DPI). The DPI is computed as follows:


(2) $$\mathrm{DPI}=\;N_{dc}/N_{Tc}\;$$

where *N_dc_* is the number of different MeSH phenotypes and biological function classes associated with the lipid, and N_*TC*_ is the total number of MeSH phenotypes and biological function classes in LipidFun.

### 2.5 Association visualization

Using the R statistical software (R version 4.0.0), we conducted an enriched network analysis with the *mwcsr* package, focusing on networks exceeding 50 phenotypes or biological functions and those containing over 100 lipids. In these networks, edges were defined based on the hierarchical structure of disease categories in the MeSH database, and nodes were weighted using the budget maximum weight connected subgraph algorithm, with their sizes and colors reflecting literature counts linking lipids and biological functions. The collapsibleTree package was also used to create an interactive tree, highlighting phenotypes or biological functions linked to specific lipids based on MeSH disease categories, where node size denotes cumulative downstream literature references. Finally, we used the *wordcloud2* package to visualize phenotypes and biological functions, with larger word sizes prominently indicating a higher frequency in the literature.

## 3 Results

LipidFun is a lipid function database and offers an extensive repository that reveals the relationships between lipids, biological functions, and diseases. Instead of only cataloging lipid entities, LipidFun provides a unique 3-fold perspective: lipids, biological functions, and the phenotypes they impact.

### 3.1 LipidFun construction

**Figure 1.**
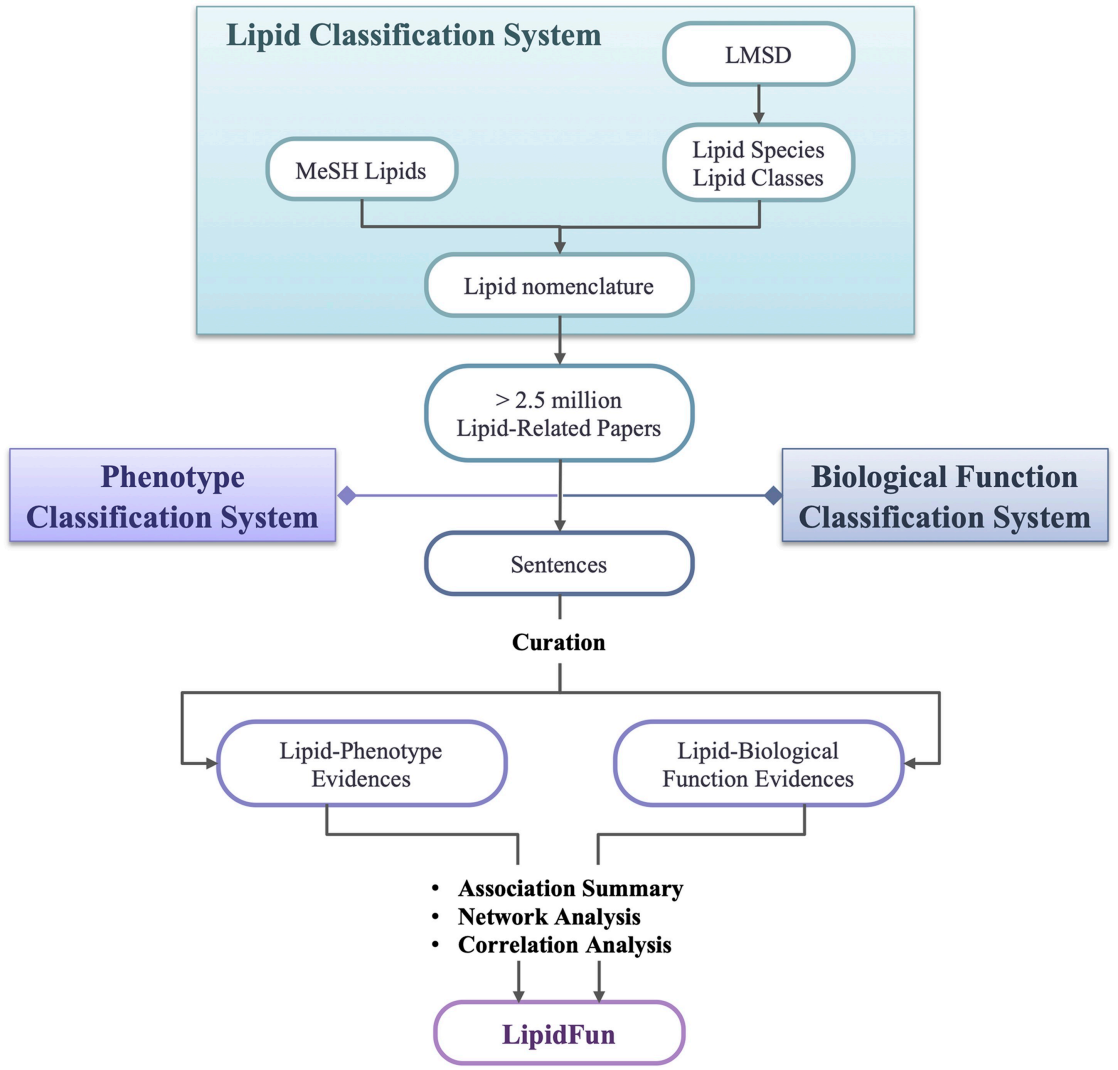
Flowchart illustrating the construction of LipidFun. This figure outlines the sequential steps involved in developing LipidFun, a comprehensive framework for lipid analysis. The flowchart delineates the process, beginning with developing the lipid classification system. It proceeds with the meticulous curation of sentence-level evidence, leading to various analytical approaches. Key databases and terminologies are denoted as MeSH: Medical Subject Headings, LMSD.

To optimize the extraction of lipid-related terms from the scientific literature (Fig. 1), we used the Lipid Maps Structure Database (LMSD) (Conroy *et al.* 2023) as the major backbone, supported by a vocabulary database, MeSH. LMSD is the largest relational database encompassing the structures and annotations of biologically relevant lipids with systematic lipid nomenclature. MeSH is a controlled vocabulary thesaurus curated by the National Library of Medicine. PubTator Central (Wei *et al*. 2013) was used as the publication source, containing 27 million abstracts and providing automated annotations for chemicals normalized to MeSH identifiers. We collected lipid-related MeSH IDs and keywords from these databases, which were then used to collect 2.5 million lipid-related articles. In order to associate lipids with phenotypes and biological functions, comprehensive databases such as PubTator, MeSH, Human Phenotype Ontology (HPO) (Köhler *et al.* 2021), and Unified Medical Language System (UMLS) (Bodenreider 2004) were used to annotate relevant terms. Table 2 lists the synonym sources and controlled vocabulary used in LipidFun. Domain experts selected sentences containing lipid terms with either phenotype or biological function terms for further pattern-based validation and manual curation. The curated sentence-level lipid–phenotype and lipid–biological relationships were then used for further network analyses, serving as the foundation for constructing LipidFun. Detailed methodologies regarding lipid phenotype, biological function definitions, sentence extraction and curation, association analysis, and visualization are provided in the Methods section.

**Table 2.** Synonym sources and controlled vocabulary used in the classification systems of LipidFun.

Name entity	Controlled vocabulary	Synonym sources
Lipid	LMSD & MeSH	LIPID MAPS Structure Database MeSH: Lipids Category
Phenotype	MeSH	PubTator: Disease Category Human Phenotype Ontology UNLS: Disease or Syndrome Category MeSH: Disease Category
Biological function	MeSH	UMLS: Natural Phenomenon or Process Category MeSH: Phenomena and Processes Category

### 3.2 The lipid classification system in LipidFun

Lipids are a diverse group of organic molecules known for their hydrophobic properties. They play crucial roles in biological processes, making it necessary to have a unified classification system to comprehensively explore their functions and implications in diseases. While previous studies, such as PubTator, have annotated MeSH terms for compounds, including lipids, in the scientific literature, MeSH is not a lipid-focused database. Therefore, a gap exists between its lipid definitions and existing lipid databases. To address this issue, we have undertaken a meticulous curation effort to harmonize lipid entities and classifications, leveraging the structured framework provided by the LMSD. The detailed information is described in the Methods section and the counts of distinct lipid classes and species curated from MeSH and LMSD are summarized in Supplementary Table S3. Our integration of MeSH terms into the LMSD nomenclature system not only allows for the systematic labeling of lipids mentioned in the literature but also facilitates their categorization based on their multifaceted structural and functional characteristics.

**Figure 2.**
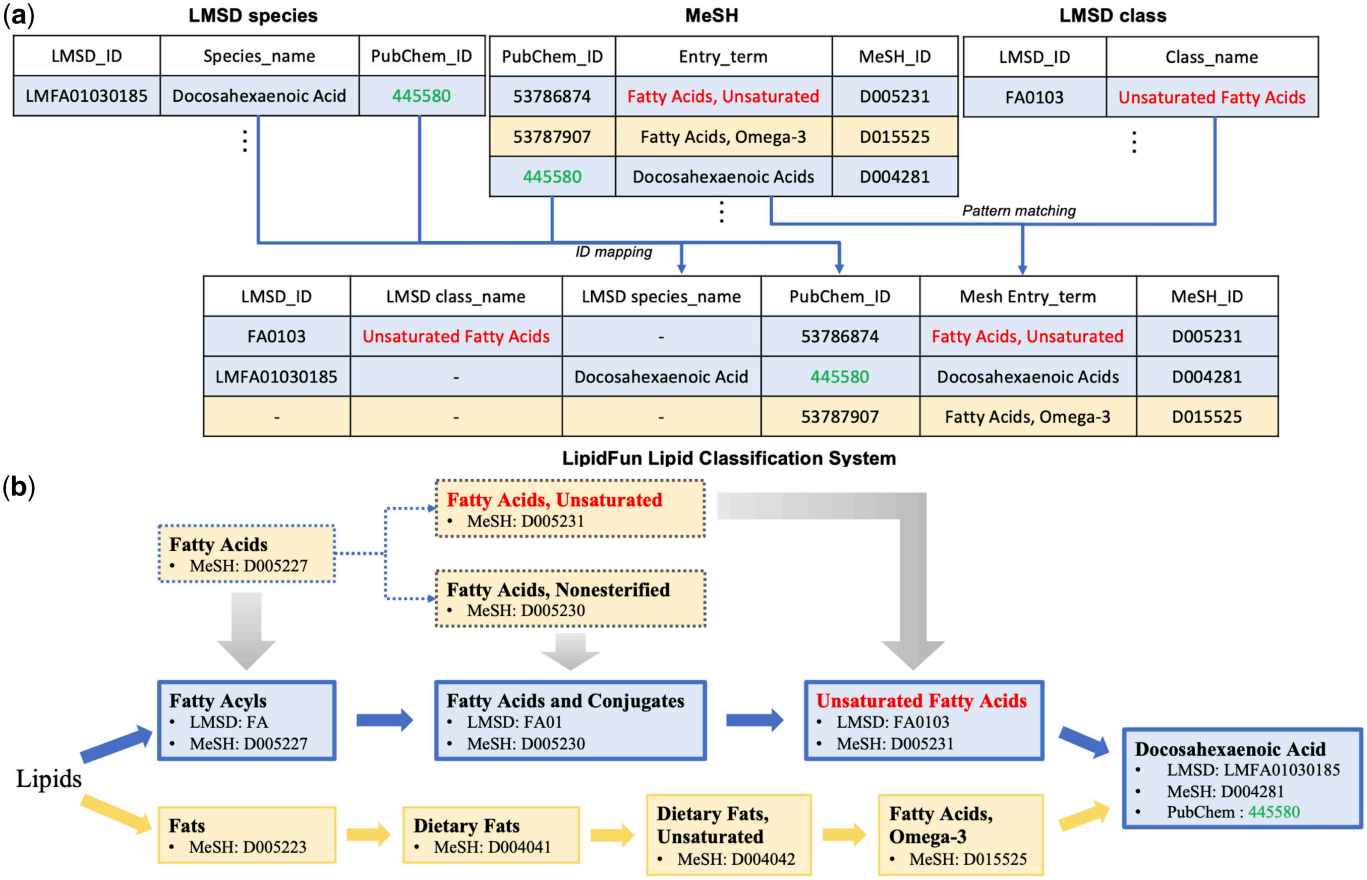
The integration of lipid nomenclature systems in LipidFun. (a) Schematic diagram illustrating the integration of the LMSD and MeSH. The figure depicts source records from both MeSH and LMSD, including lipid classes and species, along with examples of integrated entries. Records eligible for integration are indicated with a blue background, while those exclusive to MeSH are shown with a yellow background. Font colors highlight specific examples: green for integrating LMSD species with MeSH and red for integrating LMSD classes with MeSH. Arrows represent the columns utilized for merging, using ID mapping or pattern matching techniques. (b) Depiction of the nomenclature system for “Docosahexaenoic Acid,” which is recorded in both LMSD and MeSH. The architecture of LMSD serves as the primary framework within LipidFun. MeSH items with identical biological meanings (upper section) are merged into the main framework (middle section) via gradient grey arrows. Examples include the merging of Fatty Acids (D005227), Fatty Acids, Unsaturated (D005231), and Fatty Acids, Nonesterified (D005230) into Fatty Acyls (FA), Fatty Acids and Conjugates (FA01), and Unsaturated Fatty Acids (FA0103), respectively, within the LipidFun nomenclature system. Conversely, MeSH items with distinct biological meanings are retained as new branches from the main framework (lower section), such as Fats (D005223), Dietary Fats (D004041), Dietary Fats, Unsaturated (D004042), and Fatty Acids, Omega-3 (D015525). Red font color indicates entries used for merging LMSD classes with MeSH, while green font color denotes entries used for merging LMSD species with MeSH. The blue background signifies records from LMSD and MeSH that can be integrated, whereas the yellow background highlights records that can only be obtained from MeSH. Solid borders of boxes in (b). represent records constituting the main framework in LipidFun, while dashed borders indicate records from MeSH that have been merged into this framework.

To demonstrate the lipid classification system construction rationale, we present a schema (Fig. 2a) illustrating the construction of the lipid classification system in LipidFun, which outlines the data workflow for merging LMSD with MeSH. Lipid species can be directly mapped to MeSH using shared PubChem IDs found in both LMSD and MeSH; for instance, the PubChem ID 445580 (Fig. 2a, green in LMSD species and MeSH) corresponds to docosahexaenoic acid (DHA). However, lipid classes in LMSD do not have assigned PubChem IDs. Therefore, we used a pattern matching approach supplemented by expert manual verification to integrate lipid classes from LMSD with MeSH, assigning appropriate MeSH IDs to these classes. For example, FA0103 in LMSD corresponds to “Unsaturated Fatty Acids,” while D005231 in MeSH refers to “Fatty Acids, Unsaturated” (Fig. 2a, red in LMSD class and MeSH).

To demonstrate the hierarchy of the lipid classification system, we takes fatty acids as an exemplify. Fatty acids are identified in the LMSD and MeSH database with different identifiers, [FA] and [D005227], respectively. Consequently, LipidFun associates the MeSH terms with the identical terms in the LMSD, such as unsaturated or nonesterified fatty acids, into a unified nomenclature system (Fig. 2b, grey arrow and dash rectangle). In addition, for multifaceted functional characteristics, we take docosahexaenoic acid (DHA) as an example. “Docosahexaenoic acid” [LMFA01030185] is classified under unsaturated fatty acids (Fig. 2b, blue rectangle) in the LMSD and cataloged as [D004281] in the MeSH database. In addition, DHA can be recognized as an omega-3 fatty acid within dietary fats (Fig. 2b, yellow rectangle). However, common names also frequently occur in the lipid-related literature, such as omega-3 in dietary or nutrition-related studies. Notably, these nonofficial lipid names are not included in the LMSD classification system, which focuses on the official nomenclature of lipids. Consequently, we added these common names, such as omega-3 in dietary fats in the MeSH, not in the LMSD, into the LipidFun classification system, thereby expanding lipid-related literature in LipidFun. The detailed information is described in the Methods section. Based on this concept, the LipidFun classification system, based on two resources (MeSH and LMSD; Fig. 1), can elucidate the multifaceted characteristics of DHA by tracing its upstream categories in both sources (Fig. 2b).

Moreover, LipidFun provides comprehensive information, including lipid IDs, alleles, synonyms, abbreviations, and chemical structures, within an easy-to-use interactive web application, as shown in Supplementary Fig. S2, enabling researchers to gain deeper insights into lipid-related findings with improved clarity and consistency.

A standardized lipid classification system is pivotal for lipid-disease and lipid–biological function analyses. It is the cornerstone for establishing consistent terminology and facilitating precise queries on specific lipid categories and their associations with phenotypes or biological functionalities. In addition, LipidFun extends its classification domains to include phenotypes and biological functions, providing enriched insights into functional dynamics and facilitating a deeper understanding of the multifaceted interrelationships among functions influenced by specific lipids. Consequently, the classification system built into LipidFun is instrumental in expanding our conceptual understanding of the diverse roles of lipids in biological systems, establishing it as a central tool for conducting exhaustive and focused studies on lipid-related diseases and functions.

### 3.3 Sentence-level evidence in LipidFun

LipidFun identifies lipid-target relationships based on sentence-level evidence. As detailed in the Methods section, these sentences underwent rigorous validation through manual curation or NLP-based techniques. Finally, as shown in Table 1, our NLP approaches identified 591 577 sentences as lipid–biological function associations and 552 238 sentences as lipid–phenotype associations with positive predicted values (PPVs) of 0.90 and 0.91, respectively (Supplementary Tables S1 and S2). Among those sentences, 3011 lipid–biological function and 3608 lipid–phenotype sentences were manually curated, as described in the Supplementary Methods. Overall, LipidFun elucidates comprehensive relationships between lipids, phenotypes, and biological functions, providing profound insights into lipids’ diverse roles and effects in biological contexts.

**Figure 3.**
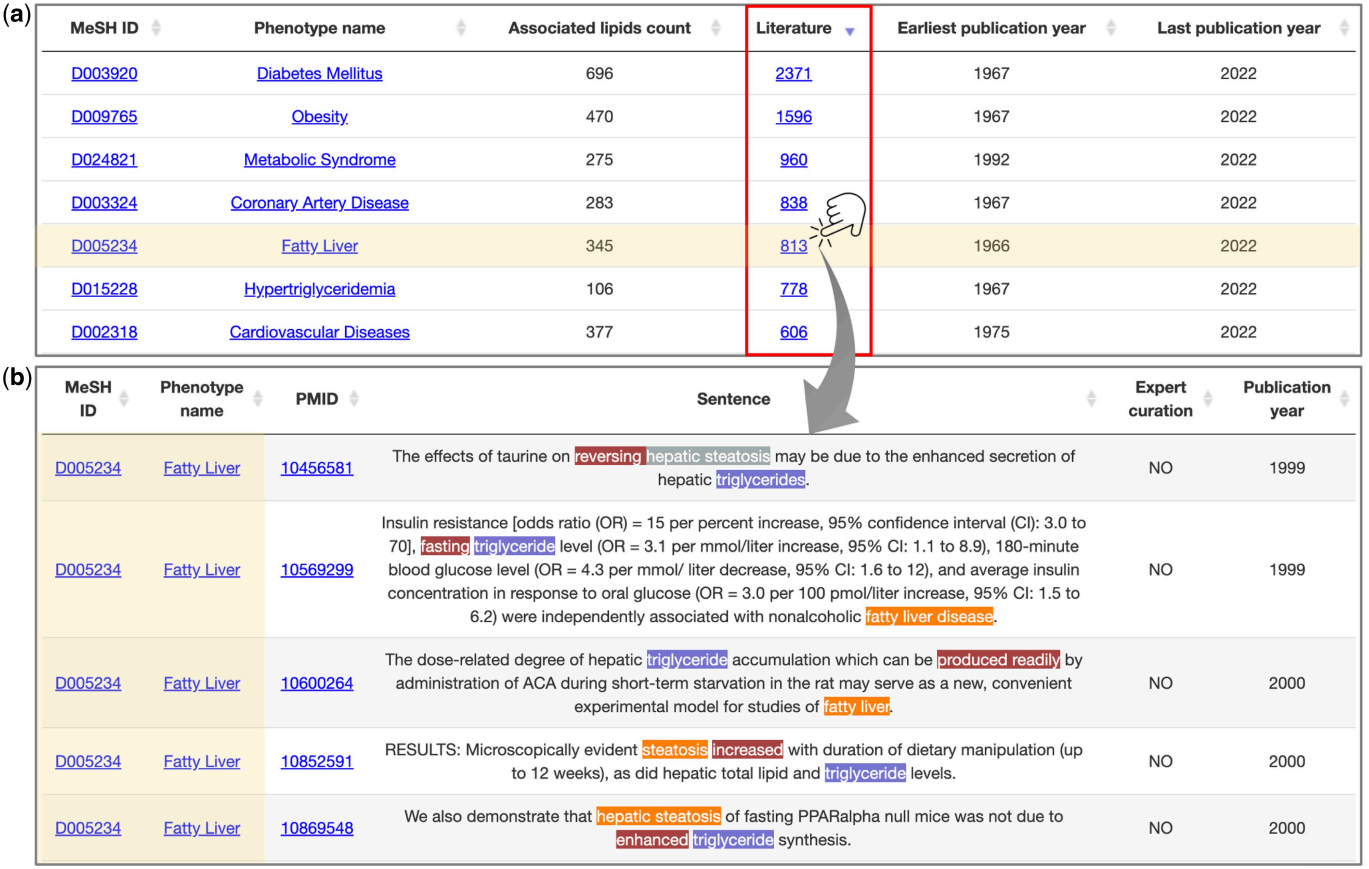
A demonstration of triacylglycerol-related phenotypes in sentence-level evidence in LipidFun. (a) Overall summary, including the number of literature (red rectangle), the publication periods, and (b) detail evidence, including name entities, PubMed ID, publication year, and manual curation confirmation status.

After the annotation and co-occurrence analysis of lipids, phenotypes, and biological functions, LipidFun is the only database currently providing sentence-level evidence for the biological functions and phenotype associations of lipids. As illustrated in Fig. 3a and Supplementary Fig. S3a, LipidFun highlights the associations between triacylglycerols (TAGs) and specific phenotypes or biological functions, complemented by metadata (e.g. publication counts and years). Additionally, detailed information, including the source articles and key sentences, is presented as illustrated in Fig. 3b. Notably, the highest-ranking, by the number of literatures, phenotypes—such as diabetes mellitus and fatty liver disease—and related biological processes (e.g. lipid metabolism) are consistent with the well-established roles of TAGs. To further enhance utility, LipidFun supports user-driven searches and enables the exploration of specific phenotypes or functions to identify corresponding lipids. For instance, when querying apoptosis, the system appropriately identifies lipopolysaccharides, ceramides (Cers), and adriamycin (Supplementary Fig. S3b). Likewise, a query on fatty liver disease reveals direct associations with cholesterol and palmitic acid (Supplementary Fig. S3c). To enrich these associations further, LipidFun introduces two indices, the disease specificity index (DSI) and pleiotropy index (DPI), which are designed to distinguish whether lipid-associated phenotypes or functions span a broad spectrum or are predominantly clustered in specific categories. For example, cholesterol is associated with 793 phenotypes spanning multiple disease categories, resulting in a low specificity index (DSI = 0.29) due to its broad implications and a high pleiotropy index (DPI = 0.96) reflecting its diverse biological roles (Supplementary Fig. S3c). In contrast, taurolithocholic acid, derived from cholesterol, is predominantly studied for its effects on cholestasis, as highlighted in most literature. This narrow focus translates to a high specificity index (DSI = 0.76) and a low pleiotropy index (DPI = 0.17) (Supplementary Fig. S3d).

### 3.4 Dual query

**Figure 4.**
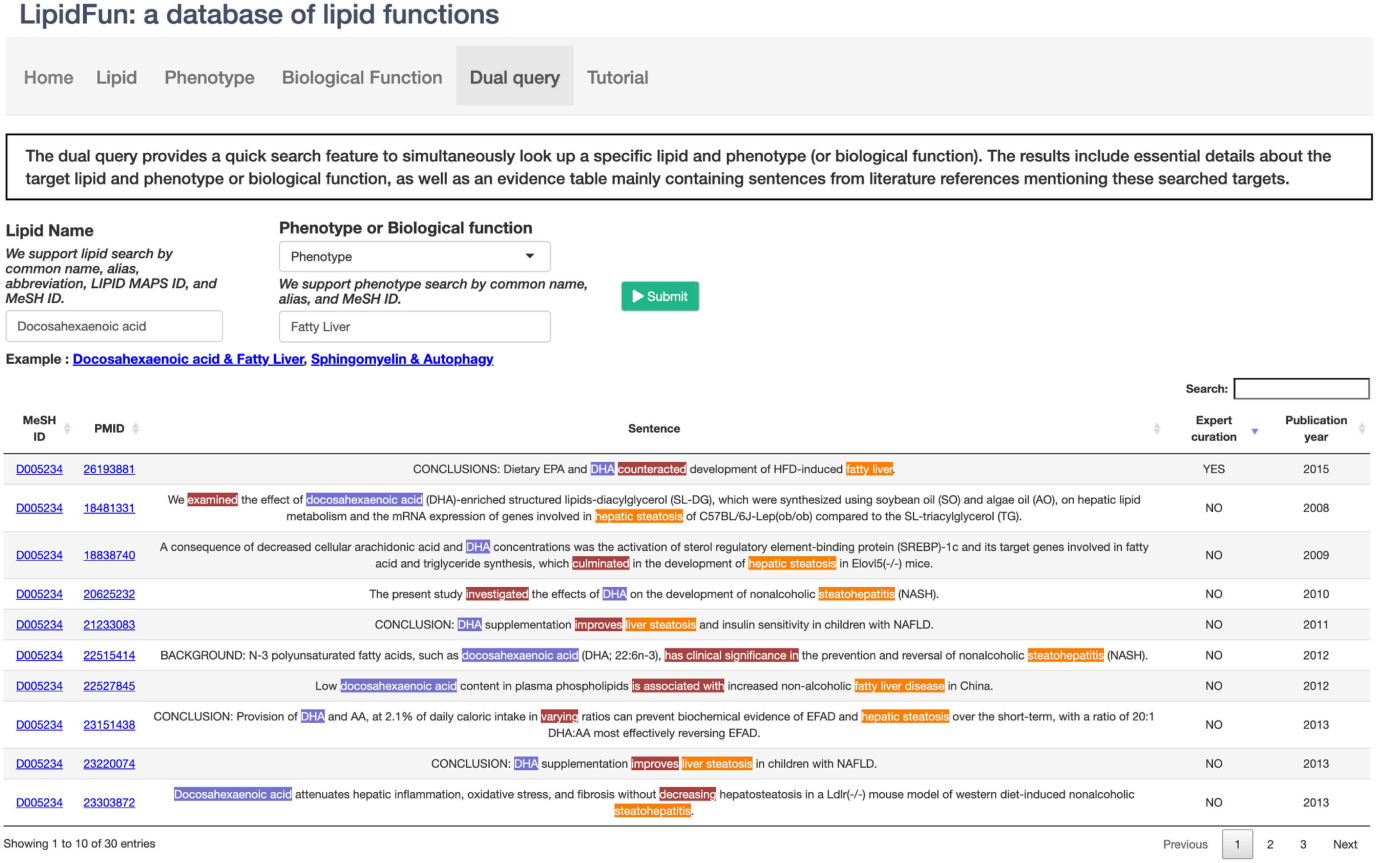
The dual query function of LipidFun. The interface of the dual query function is demonstrated. Association type, “Phenotype” and “Biological function,” can be selected in the Select type section (middle left). By providing lipid name and phenotype/biological function name in the query boxes (middle), respectively. Sentence-level evidences of the lipid-related phenotype or biological function will be shown and the corresponding name entity are highlighted for clarity (lipid: blue, phenotype/biological function: light brown, and verb: dark brown).

To enhance user accessibility and expedite queries within LipidFun, we have developed a “Dual query” function (Fig. 4). This functionality enables users to simultaneously search for associations between lipids and either phenotypes or biological functions, facilitating quicker access to relevant results. For example, when exploring lipid–phenotype relationships, a user can query both “docosahexaenoic acid” and “fatty liver,” allowing for the rapid identification of their association, such as the finding that dietary DHA counteracts the development of HFD-induced fatty liver (Soni *et al.* 2015). Similarly, when investigating lipid–biological function, a user can query both sphingomyelin and autophagy, leading to prompt identification of their association, such as the discovery that poliomyelitis enhances autophagy (Cao *et al.* 2019).

### 3.5 Association analysis and visualization in LipidFun

The combination of classification systems and a large amount of sentence-level evidence allows us to gather evidence of downstream entities for the upstream ones. This enables us to merge numerous lipid-target associations into a comprehensive visualization, including Network View, Inferred Tree View, and Word Cloud View, providing an overview of the associations.

#### 3.5.1 Network view

**Figure 5.**
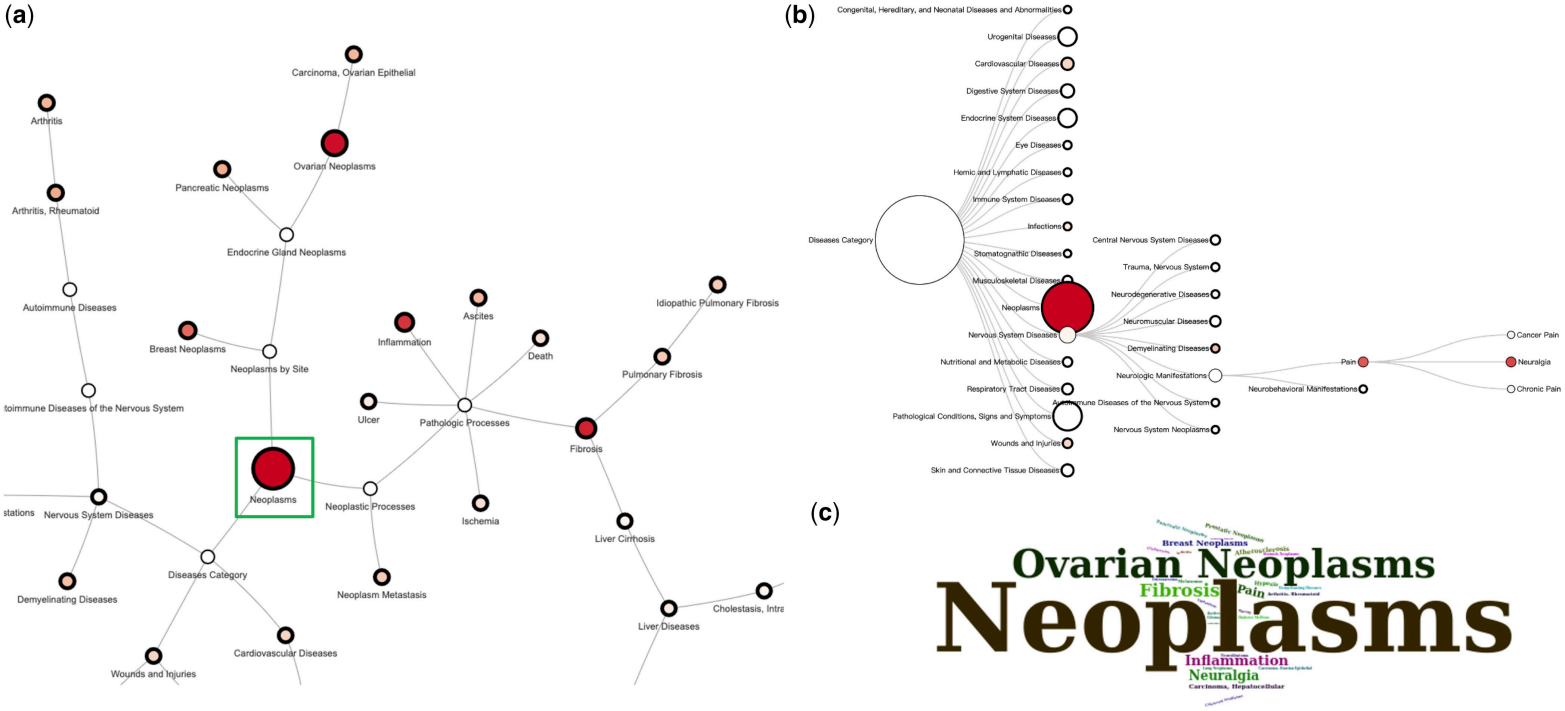
Visual depictions of phenotypes associated with lysophosphatidic acid. This figure offers a multifaceted visual representation of phenotypes related to lysophosphatidic acid, utilizing various data visualization techniques. (a) Network view: the network diagram depicts the connections between the target lipid and its associated phenotypes. Each node's size and color gradation signify the volume of literature documenting the lipid–phenotype association, with larger and redder nodes indicating a more significant number of records. Nodes lacking literature support are marked in white. The green rectangle highlights the phenotype, neoplasm, mentioned in the main text. (b) Inferred Tree View: This view presents a hierarchical organization of phenotypes based on disease categorization in MeSH. The tree structure elucidates the hierarchical relationships among phenotypes within specific disease categories. The size of each node reflects the aggregate literature count of its subordinate nodes, and the node color corresponds to the individual literature count, transitioning from white (no literature) to red (substantial literature). Bold-framed nodes denote those with subsequent lower-level nodes. (c) Word Cloud View: The word cloud format illustrates the prevalence of phenotypes associated with the target lipid, where the prominence of phenotype names correlates with the extent of literature evidence, with larger font sizes indicating a higher frequency of associated literature documentation.

LipidFun merges myriad lipid-target associations into a comprehensive network, greatly enriching the visualization and understanding of intricate relationships between lipid, phenotype, and biological functional clusters. The lysophosphatidic acid-associated phenotype network is shown in Fig. 5 to demonstrate the concept. Figure 5a highlights the significant association between lysophosphatidic acid and neoplasms (Fig. 5a, green highlight rectangle), with a pronounced focus on ovarian cancer, while also drawing links to breast and pancreatic cancer.

The versatility of LipidFun is further highlighted by its ability to drill down into specific phenotypes or biological functions, allowing users to map the associated lipid networks. For example, adipogenesis, a process that converts fatty acids into TAGs for storage, has a clear association in the adipogenesis-associated lipid network (Supplementary Fig. S4a).

#### 3.5.2 Inferred tree view

LipidFun extends beyond the traditional network to an insightful inferred tree view that aggregates association results within the scope of the lipid classification system. This unique feature allows users to delve deeper into the precise upstream categories to which the affected lipids are assigned. As shown in Fig. 5b, the tree view for lysophosphatidic acid reinforces previous findings by highlighting major affected disease categories such as neoplasms, nervous system diseases (e.g. neuralgia), respiratory diseases (e.g. pulmonary fibrosis), and various other pathological conditions, signs, and symptoms. As we explore the lipid tree structure for adipogenesis (Supplementary Fig. S4b), we observe its influence on well-known lipid classes such as fatty acyls and glycerolipids. Furthermore, the tree structure highlights some interesting and well-established correlations, specifically the effects of prenol lipids and polyketides on adipogenesis, exemplified by retinoids and flavonoids, respectively (Supplementary Fig. S4b), which are also correlated to the Network View (Supplementary Fig. S4a).

#### 3.5.3 Word cloud view

LipidFun incorporates a word cloud as part of its visualization strategy to highlight the frequency and significance of key associated features, providing an immediate snapshot of the most pertinent elements (Fig. 5c and Supplementary Fig. S4c). In essence, LipidFun’s suite of visualization methods captures the intricate dynamics between lipids, phenotypes, and biological functions. By presenting this synthesized data, even nonlipid specialists can quickly grasp the prevailing research landscape of specific lipids.

## 4 Discussion

LipidFun represents a pioneering lipid function database, merging the nuances of lipid classification with the intricate connections between lipids and distinct phenotypes or biological functions. To achieve this, LipidFun meticulously integrates data from respected sources, LipidMaps and MeSH, crafting a robust and hierarchically stratified lipid classification system. Such integration empowers the extraction and systematic categorization of lipid-centric literature, laying the groundwork for subsequent network analyses. By leveraging the MeSH-controlled vocabulary, we create intricate connections between databases such as PubTator, HPO, and UMLS. Crucially, MeSH IDs harvested were used to extract lipid-related biological functions and phenotypes from the PubTator database, forming the foundation of our curation. Furthermore, the reliability of LipidFun’s data is ensured through a combination of domain expertise and NLP techniques, ensuring high-quality curation of lipid-related information. Our visualization tools distill lipid relationships into intuitive word clouds, enriched networks, and detailed tree views, facilitating swift data interpretation. Overall, LipidFun aims to elucidate hidden mechanisms that may transform our understanding of lipid-related phenotypes and biological functions.

LipidFun differentiates itself from other contemporary tools, such as LipidPedia and LipiDisease, by incorporating a unique lipid classification system. This system organized lipids into a hierarchy based on MeSH and LMSD, allowing for systematic classification of lipid, including class and species. Additionally, LipidFun is the sole platform that provides sentence-level evidence, which elucidates the intricate interconnections between lipids, phenotypes, and biological functions. This feature enhances the understanding of how specific lipids influence phenotypic expressions and biological activities. Furthermore, 6619 lipid-function related sentences had been curated by 4 experts manually. Last, LipidFun offers an extensive array of visualization tools that illustrate lipid-related functions from multiple perspectives, thereby enriching the analysis of lipid biology.

Instead of only assess the co-occurrence of entity pair in the sentence, the large language model was applied to assist in extracting meaningful associations and more than 7000 sentences are further confirmed manually by four domain experts. The NLP-curated sentences are underscored by impressive PPVs of 0.90 and 0.91 for lipid–biological function (Supplementary Table S1; TP = 2635, FP = 298) and lipid–phenotype association (Supplementary Table S2; TP = 2893, FP = 301), respectively. Our results show that the NLP approach can achieve a high PPV while maintaining moderate sensitivity, 0.87 for lipid–biological function and 0.80 for lipid–phenotype association, as the trade-off in recognizing lipid function. Consequently, while we cannot fully identify all associations, the quality of the identified sentences in LipidFun is promising due to the high precision. The high accuracy of association identification may be due to the co-occurrence of lipid and phenotype or biological function within one sentence of the abstract, which is the most biologically or medically meaningful conclusion of the scientific study summarized by the authors. Consequently, when focusing on associations between lipids and phenotypes or biological functions, the filtered co-occurrence sentences remain accurate, suggesting that the curation offered by the NLP might be supplementary.

LipidFun is currently designed to curate associations derived from sentences in existing published studies rather than to predict novel lipid-related associations. Consequently, all associations reported in LipidFun are rooted on current literatures, most of which are substantiated by experimental results. Leveraging this framework, the association reported by LipidFun can be evaluated by the number of supporting literatures (Fig. 3a, red rectangle) and assessed directly by inspecting the corresponding sentences (Fig. 3b). Furthermore, to augment user comprehension at the sentence level, we use a color-coded evidence table that highlights entities and verbs across data sources (Fig. 3b). Accompanied by a summary chart, this table contains counts of associated phenotypes, biological functions, pertinent literature citations, and key indices. Given our meticulous approach and intuitive visualizations, we are confident that users will find LipidFun invaluable in decoding the nuances of lipid-associated biological functions and phenotypes.

A unique feature of LipidFun is its classification system of lipids, biological functions, and phenotypes, which allows it to provide an overview of lipid–phenotype and lipid–biological function associations (Supplementary Figs S4 and S5). To accomplish this, we used MeSH as the classification system for biological functions and phenotypes. While the LMSD is considered the gold standard for lipid classification systems, current resources such as Pubtator annotate compounds, including lipids, in the literature using MeSH terms. Integrating the LMSD with MeSH terms into LipidFun represents a strategic advance, broadening lipid annotation coverage and anchoring it within the universally respected framework of contemporary lipid research (Supplementary Table S3). Crucially, LipidFun’s lipid classification system standardizes terminologies (Table 2), streamlining precise lipid inquiries and unraveling their interconnected upstream and downstream relationships (Fig. 2), which is especially pivotal since lipids with similar structures usually traverse common metabolic pathways, implying synchronous fluctuations in their levels. Instead of an isolated perspective on distinct lipid species, our network analysis assimilates data holistically, offering a panoramic view of lipid alterations influenced by specific biological functions or phenotypes and highlighting their categorical affiliations (Fig. 5b and Supplementary Fig. S4b). Such an encompassing approach not only reveals the intricacies of lipid reprogramming but also identifies avenues for probing imbalanced lipid pathways and enzymes. Similarly, LipidFun’s phenotype and biological function network provides lipids with functional annotations derived from contemporary research, elucidating their roles in biological matrixes (Fig. 5a and Supplementary Fig. S4a). This interlinked network becomes an incubator for hypothesis generation, directing attention to those intertwined biological functions or phenotypes that, through existing research, show tangible ties to particular lipids, priming them for experimental validation.

While the total number of associations in LipidFun—591 577 for biological functions and 552 238 for phenotypes—is lower than that in LipidPedia (1 553 125), LipidFun annotates a greater number of unique lipids (2490) compared to LipidPedia (875), as illustrated in Supplementary Fig. S6. This discrepancy can be attributed, in part, to the broader annotation perspectives adopted by LipidPedia, which include associations with genes and cellular locations, aspects that are not currently annotated in LipidFun. Additionally, the methodologies underlying the two databases differ significantly. LipidPedia utilizes text-mining technology at the document level to extract lipid-related information without pinpointing specific sentences that detail these associations. In contrast, LipidFun ensures that every association is directly supported by corresponding sentences, thereby providing a more granular and rigorous level of evidence compared to LipidPedia.

In the future development of LipidFun, the database will be expanded by incorporating a larger number of manually curated sentences to enhance the robustness and quality of evidence for lipid-related associations. Additionally, the system’s NER capabilities for phenotypes and biological functions will be improved through the integration of advanced text-mining tools, such as PhenoTagger (Luo *et al.* 2021), to broaden the scope and increase the accuracy of entity identification. Furthermore, the use of increasing curated data, combined with advanced large language models, will be used to further enhance the precision of the system.

## Supplementary Material

btaf110_Supplementary_Data

## Data Availability

LipidFun is freely available: https://lipidfun.bioinfomics.org/.
