# Supplementary material for: LipidFun: a database of lipid functions
Source: Bioinformatics. 2025 Mar 13;41(4):btaf110. doi: 10.1093/bioinformatics/btaf110 (PMC11974499; doi:10.1093/bioinformatics/btaf110)
Supplement: btaf110_Supplementary_Data [file btaf110_supplementary_data.docx]

**Supplementary Methods**

**Prompt**

The prompt used to identify associations between lipid-phenotype and lipid-biological function is as the following:

- You are a linguist to precisely determine whether there is an association between two terms, lipid term and function term, in the sentence based on their context, semantic meaning, and syntactic structure given in the following. Response should be limited to either 'Yes' or 'No'.
  The sentence: [a sentence having lipid name entity and function name entity]
  The lipid term: [a lipid name entity]
  The function term: [a function name entity]

**Sentence selection approach for curation**

To evaluate the reliability of associations, we randomly selected sentences based on Lipid-Biological Function and Lipid-Phenotype pairs, allowing for up to three instances for each pair. we employed the following methodological approach:

1. We first analyzed distinct lipids co-occurring with phenotypes or biological functions, resulting in distinct 2,433 lipids with phenotypes and 1,701 lipids with biological functions.
2. Subsequently, we selected up to three sentences for each distinct lipids associated with phenotypes and with biological functions, respectively. This process yielded 4,095 sentences for lipid-phenotype and 3,453 sentences for lipid-biological function, a total of 7,548 sentences out of 6,463 abstracts, for manual assessment.
3. Finally, after the manual curation process, 3,608 lipid-phenotype sentences covering 2,211 distinct lipids and 3,011 lipid-biological function sentences covering 1,581 distinct lipids were curated.

This approach enables a comprehensive assessment of all potential associations during the manual curation process conducted by four domain experts. Our selection process was randomized across the entire dataset, without restricting it to any specific subset of abstracts.

**Manual curation process**

Totally 4 experts with biology and medical research background are devoted into the manual curation task of judging the lipid-phenotype and lipid-biological function association in the sentence. Only if the association was confirmed by all experts independently, then it can be regarded as Expert Curation (as shown in Figure 2). Consequently, based on these manually confirmed data, we can evaluate the performance of NLP in association identification (Supplementary Table 2 and 3).

An online web system was constructed for the manual curation task to help experts and to make sure the independency of the judgment making of the experts. The name entities of the lipid name entity, the target name entity, and the relationship entity were highlighted in the system and experts should use their expertise to judge whether the lipid name is associated with the target name. These manual curation activities of experts were recorded within a database and results of the independent judgement were analyzed.

**Annotation guideline of the lipid-oriented relations task**

*Task Definition*

The primary objective of the " Annotation Guideline of Lipid-Oriented Relations" is to annotate the sentence-based relationships between lipids and their biological functions as well as their associations with various phenotypic outcomes. This annotation task plays a crucial role in advancing our understanding of the intricate connections between lipids and the broader biological processes they impact.

*Entity Definition*

Three primary types of entities will be labeled: lipid entities, biological function (BF) entities, and phenotype (PH) entities. These entities will be pre-tagged within the given text to assist annotators in identifying and determining the relationships between them.

Lipid entities will be derived from a combination of reliable sources, including the Lipid Maps, the MeSH lipid subtree, the UMLS T119 subtree (Lipid), and the swissLipids database. These sources provide comprehensive coverage of various lipid molecules, ensuring a broad representation of lipid entities in the annotation process.

Biological function (BF) entities will be labeled using information obtained from the MeSH Phenomena and Processes Category (G) and the Gene Ontology (GO) within the UMLS T70 subtree (Natural Phenomena or Process). This enables the inclusion of diverse biological functions associated with lipids and their impact on biological systems.

Phenotype (PH) entities will be identified using a combination of resources, including PubTator disease annotations, MeSH disease annotations, the Human Phenotype Ontology (HPO), and the UMLS T047 subtree (Disease or Syndrome). This comprehensive collection of resources ensures the coverage of various phenotypic outcomes and disease-related manifestations in the annotation process.

By employing a wide range of reliable sources for entity labeling, the entity definition provides a foundation for annotators to identify these key entities within the given text, facilitating the subsequent annotation of the lipid-oriented relations.

*Relation Determination*

The crucial task assigned to the curators is to assess the presence of associations between lipid entities and either biological functions (BF) or phenotypes (PH) within the context of single sentence itself. In addition to considering simple direct relationships between relation pairs, the annotations also encompass broader associations where one entity serves as a mediator or experimental method, among

others. The primary objective is to determine whether there is a potential relationship between the mentioned entities, which can be categorized into three distinct tags: "Yes," indicating the presence of an association; "NO," signifying the absence of an association; and "unknown," denoting cases where the sentence lacks sufficient information or clarity to ascertain the existence of any relationship.

*Disambiguation Principle*

To facilitate the annotation process and enhancing annotation consistency, this principle is specifically designed to address common ambiguities encountered while annotating lipid-related texts. These ambiguities fall into four main categories: Entity ambiguation, Domain ambiguation, Narrative ambiguation, and Semantic ambiguation. In the following sections, each of these categories will be introduced with illustrative examples to ensure a clear understanding of how to tackle and resolve such challenges effectively.

*Entity ambiguation*

Entity ambiguation arises from two scenarios: entities nested within gene names and incomplete pre-tagging or misplaced entities for Biological Function (BF) or Phenotype (PH) due to polysemy. When entities belong to subcategories of tags (e.g., "breast cancer" as a type of "cancer"), relationship labels are contextually assigned. However, nested names and misplaced entities lack clear relationships and are categorized as "unknown," ensuring annotation consistency and accuracy. Besides, the “synthesis,” “pathway,” and “metabolite” of certain lipid component can be regarded as the lipid itself to reduce further identification. The following instances illustrates these scenarios.

Instance:

“***Farnesyl diphosphatelipid*** *synthase attenuates paclitaxel-induced apoptotic* ***cell deathBF*** *in human glioblastoma U87MG cells.*” – “*Farnesyl diphosphate*” is covered by gene name, **unknown** relation

“*Treatment of HMDM with* ***alpha-tocotrienollipid*** *or FeAOX-6 enhanced also* ***tumor necrosisPH*** *factor-alpha secretion.*” – “*tumor necrosis*” nested within gene name, **unknown** relation

“*The* ***interphaseBF*** *proteins were replaced with* ***lecithinlipid*** *in a dose-dependent manner.*” – “*interphase*” represented a cell cycle phase by entity labeling, **unknown** relation

“***HCAlipid*** *also largely prevented cell* ***deathsPH*** *caused by a severe oxidative stress.*”

– “*death*” cannot replace “*cell death*” which is also called as apoptosis, **unknown** relation

“*Mechanistically,* ***thromboxane A2lipid*** *(TXA2) pathway was identified as the relevant molecular target for aspirin in* ***anoikisBF*** *sensitization.*” – “*pathway*” is treated as an extension of “*thromboxane A2,*” **Yes** relation

*Domain ambiguation*

Domain ambiguation is prevalent in sentences comparing experimental results, often accompanied by descriptions of inferior or insignificant effects. However, as long as the sentence does not explicitly state "no effect," even weaker effects should be considered as a relationship and classified as "Yes." Moreover, the experiments are conducted between the relation pair in the sentence description with terms such as “evaluation,” “analysis” or “examination.” Although the effectiveness is not presented in the contents, it still builds up a temporary association which can be annotated as "Yes." The following instances illustrates the scenario.

Instance:

“*Granulosa* ***cell aggregationBF*** *and aromatase activity were inhibited by phorbol ester but not completely by* ***diacylglycerollipid****.*” **– Yes** relation

“*RESULTS:* ***Cell proliferationBF*** *was promoted more in the FK506 groups than the control or* ***CsAlipid*** *groups on days 3 and 7.*” **– Yes** relation

“*Prompted by these data, we evaluated the novel* ***parthenolidelipid*** *analog, LC-1, in 54* ***CLLPH*** *patient samples.*” – **Yes** relation

*Narrative ambiguation*

Narrative ambiguation comprises two scenarios. The first involves entity pairs described separately in the sentence, such as enumerations or individual explanations, leading to a "No relation" between the pair. The second scenario entails indirect associations, where the descriptions of the entity pair involve temporal order, simultaneous observations, or non-direct causal relationships. Despite lacking a direct and explicit connection, these indirect associations are considered to have a relationship and are categorized as "Yes." The following instances illustrates these scenarios.

Instance:

“*BraLTP1, a lipid transfer protein gene involved in epicuticular* ***waxlipid*** *deposition,* ***cell proliferationBF*** *and flower development in Brassica napus.*” – enumeration, **NO** relation

“***PainPH*** *intensity was decreased, and* ***SEAlipid*** *levels were increased at the six- month follow up.*” – observation, **Yes** relation

*Semantic ambiguation*

Semantic ambiguation arises from semantic uncertainties in the text, often encountered when discussing unclear or poorly understood biological mechanisms, indicated by phrases like "unclear," "little is known," or "not well understood." Even if there might be a potential relationship in the subsequent context, the relation for such sentences should be marked as "unknown" based on the single-sentence judgment. The following instances illustrates these scenarios.

Instance:

“*However, the effects of* ***PDXlipid*** *on* ***inflammationPH*** *in adipocytes remain ambiguous.*” – “*ambiguous*”, **unknown** relation

“*However, it is unknown if* ***Baicalinlipid*** *influences* ***autophagyBF*** *after TBI.*” – “*unknown*”, **unknown** relation

**Supplementary Tables**

**Supplementary Table** **1.** The independent dataset of evaluating the performance of NLP on the lipid-biological function association identification task

| N = 3453 sentences | | Manual curation confirmed | |
| --- | --- | --- | --- |
|  |  | Yes^$^ | No |
| GPT model prediction | Yes | 2,635 [TP] | 298 [FP] |
|  | No | 376 [FN] | 144 [TN] |
| Positive predict value, [TP]/[TP+FP] = 0.90  Sensitivity, [TP]/[TP+FN] = 0.87  ^$^A total of 3,011 sentences with lipid-biological function association confirmed by manual curation. | | | |

**Supplementary Table 2.** The independent dataset of evaluating the performance of NLP on the lipid-phenotype association identification task

| N = 4095 sentences | | Manual curation confirmed | |
| --- | --- | --- | --- |
|  |  | Yes^$^ | No |
| GPT model prediction | Yes | 2,893 [TP] | 301 [FP] |
|  | No | 715 [FN] | 186 [TN] |
| Positive predict value, [TP]/[TP+FP] = 0.91  Sensitivity, [TP]/[TP+FN] = 0.80  ^$^A total of 3,608 sentences with lipid-phenotype association confirmed by manual curation. | | | |

**Supplementary Table 3.** Summary of distinct lipid class and species from two databases, LMSD and MeSH, in LipidFun. Only the lipid found within at least one sentence in LipidFun would be counted.

| Lipid source and category | Total^#^ | Both^*^ | LMSD-only | MeSH-only |
| --- | --- | --- | --- | --- |
| Fatty Acyls (FA) | 664 | 586 | 36 | 42 |
| Glycerolipids (GL) | 22 | 16 | 6 | 0 |
| Glycerophospholipids (GP) | 76 | 49 | 21 | 6 |
| Sphingolipids (SL) | 29 | 16 | 8 | 5 |
| Sterol Lipids (ST) | 308 | 281 | 19 | 8 |
| Prenol Lipids (PR) | 242 | 202 | 39 | 1 |
| Saccharolipids (SL) | 2 | 1 | 1 | 0 |
| Polyketides (PK) | 577 | 543 | 33 | 1 |
| Fats | 22 | 4 | 0 | 18 |
| Glycolipids | 16 | 8 | 2 | 6 |
| Lipopeptides | 3 | 0 | 0 | 3 |
| Lipopolysaccharides | 2 | 1 | 0 | 1 |
| Membrane Lipids | 195 | 40 | 37 | 18 |
| Oils | 27 | 3 | 0 | 24 |

^#^ Total: total lipid class and species in LipidFun

^*^ Both: lipids in both LMSD and MeSH


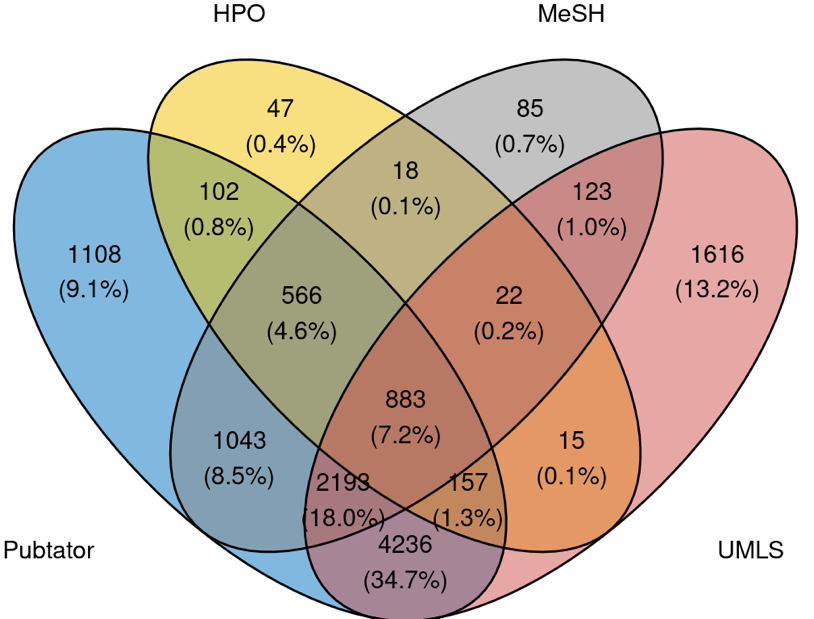


**Supplementary Figure 1.** **A Venn diagram demonstrate the overlapping name entity of four difference sources, PubTator, HPO, MeSH, and UMLS, of phenotype dictionary.** Both number of name entity and percentage in the whole dictionary are shown.

**
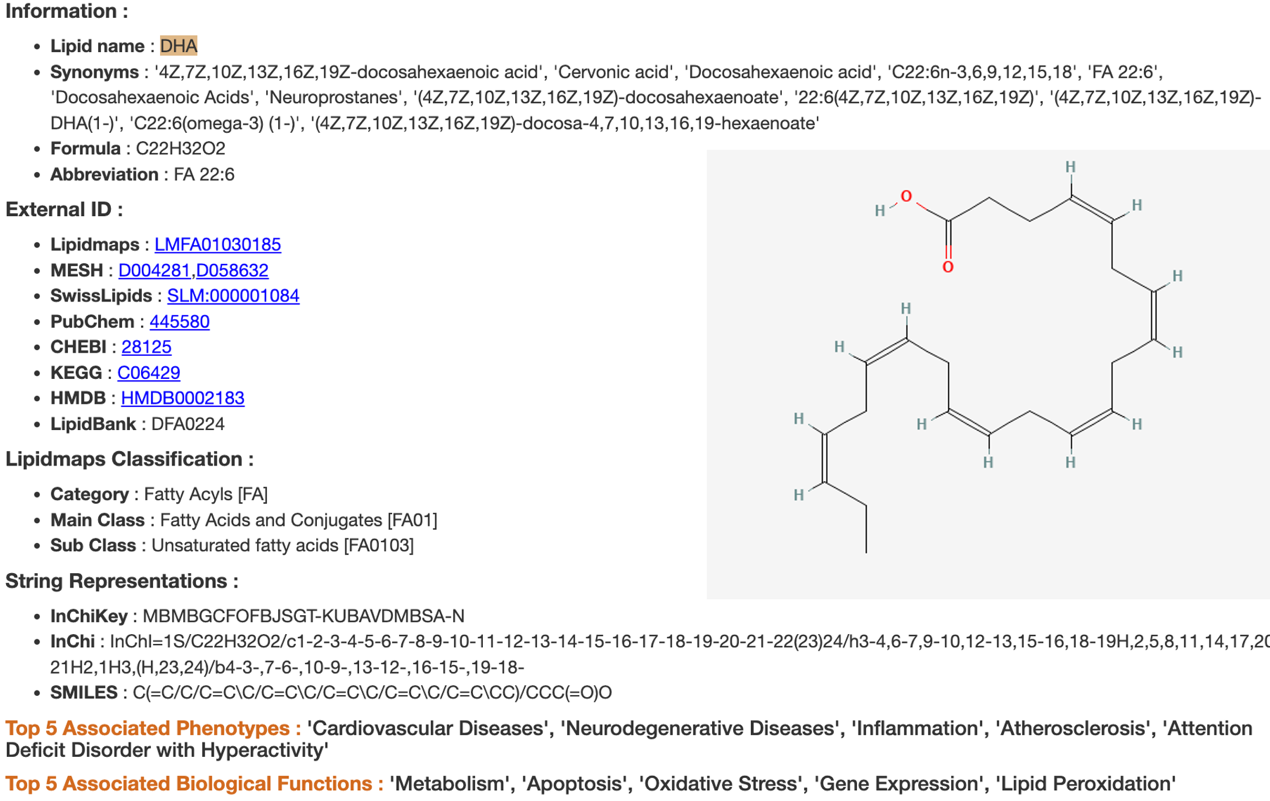
**

**Supplementary Figure 2.** **LipidFun provides an easy-to-use interactive web application for researchers to explore lipid-function associations.** At the homepage, our website allows users to search for association results using common names, aliases, abbreviations, or database IDs based on lipids, phenotypes, or biological functions. The website layout of each aspect includes a general section (Left) that displays basic information, external IDs, and the most significantly associated features for each entry, followed by detailed association results (Right) for the corresponding biomedical aspects. To provide an overview of the association results, we have also listed basic information, external ID, classification in LMSD, and the top 5 associated phenotypes and biological functions in the general section.

**
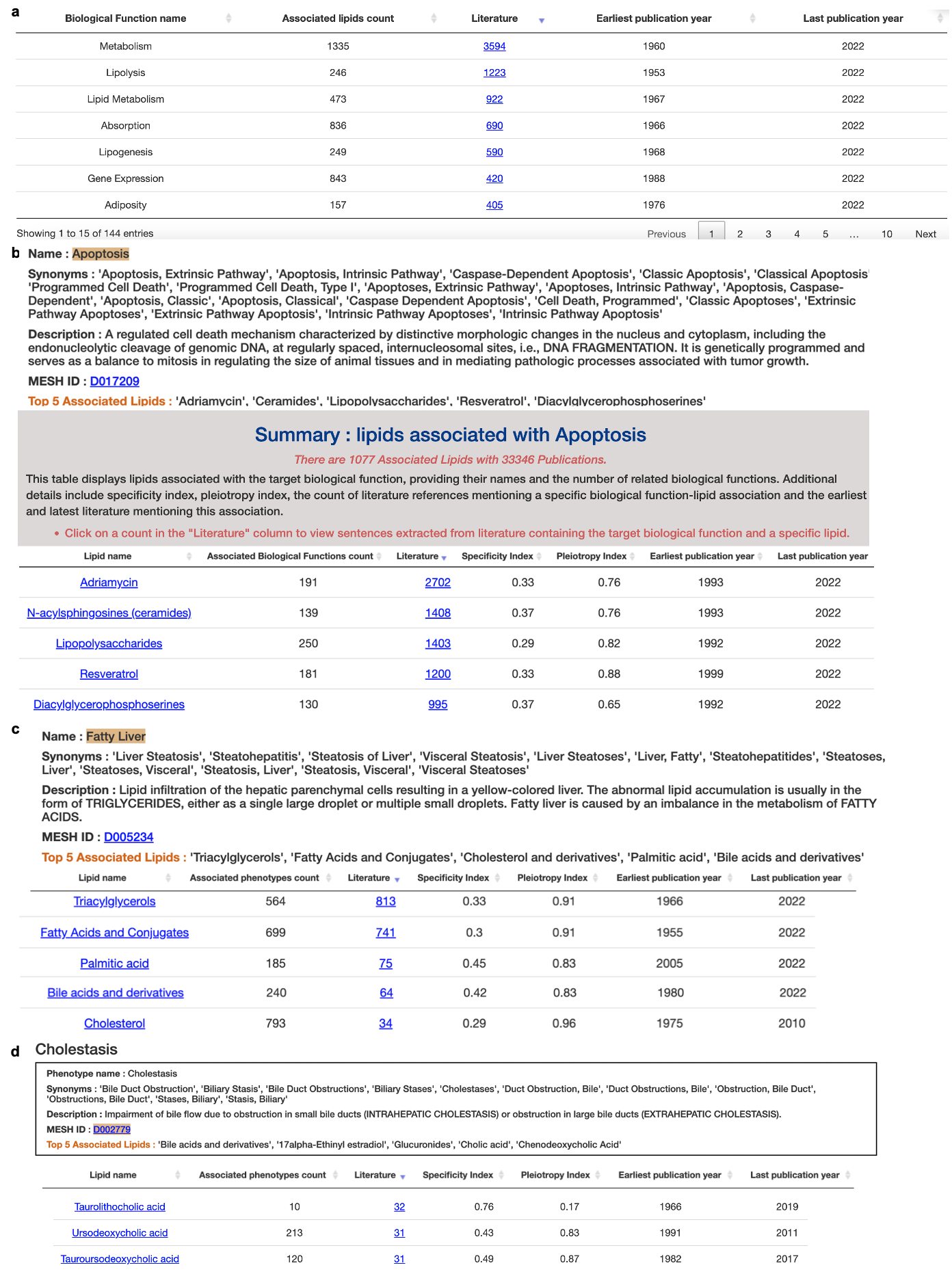
**

**Supplementary Figure 3.** A **demonstration of triacylglycerol-related biological function evidence and cholestasis-related lipid in sentence-level in LipidFun.** Results of triacylglycerol-related biological function with evidences from literatures are depicted in **a** overall summary, including the number of literatures and the publication periods. Furthermore, to extend the utility, LipidFun allows users to reverse the search and query specific phenotypes or functions, such as apoptosis in **b** and fatty liver in **c**, to identify affected lipids of relevance. Lipids associated with cholestasis were demonstrated in **d**.


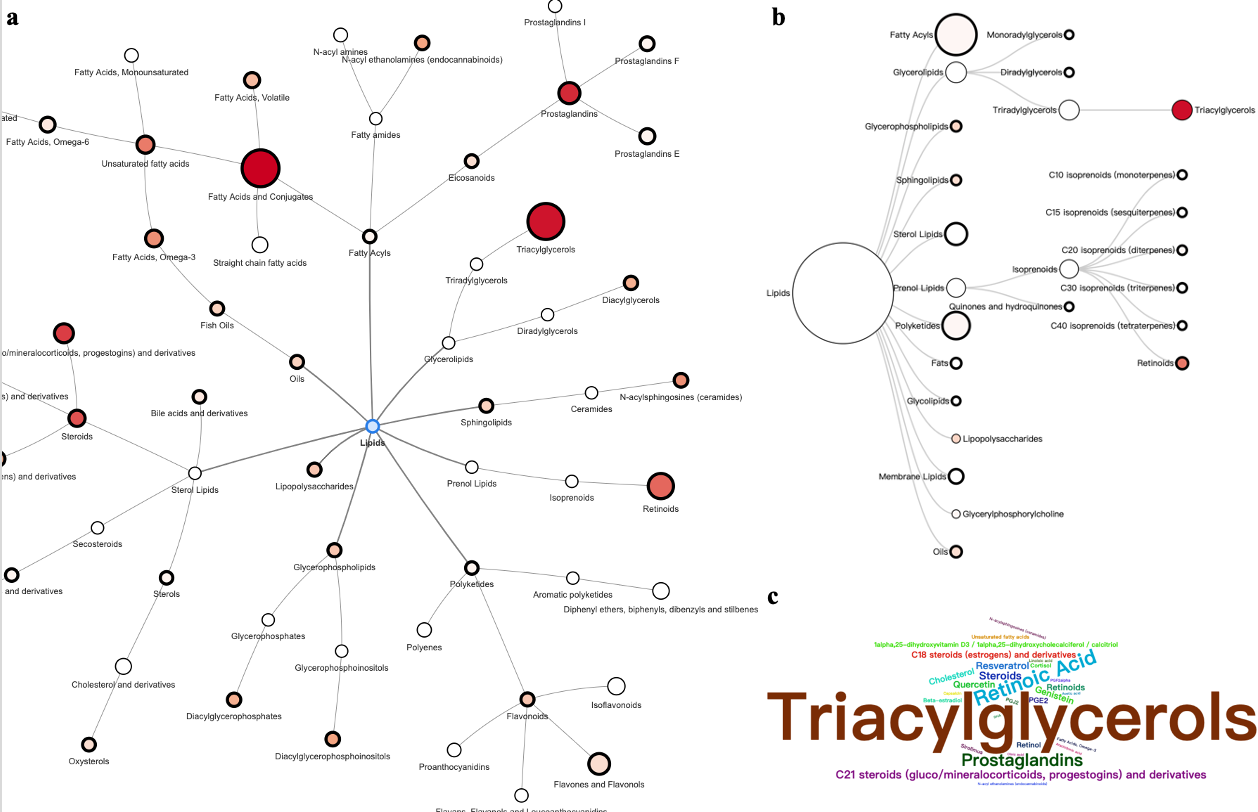


**Supplementary Figure 4. Visual Depictions of Lipids Associated with Adipogenesis.** LipidFun offers a multi-faceted visual representation of adipogenesis-related lipids, utilizing various data visualization techniques. **a** Network View: The network diagram depicts the connections among adipogenesis-related lipids. The size and color gradation of each node signify the volume of literature documenting the lipid-phenotype association, with larger and redder nodes indicating a greater number of records. Nodes lacking literature support are marked in white. **b** Inferred Tree View: This view presents a hierarchical organization of lipid based on the classification system in LMSD (LIPID MAPS Structure Database). This structure can elucidate the hierarchical relationships among adipogenesis-related lipids according to LMSD categories. The size of each node reflects the aggregate literature count of its subordinate nodes, and the node color corresponds to the individual literature count, transitioning from white (no literature) to red (substantial literature). Bold-framed nodes denote those with subsequent lower-level nodes. **c** Word Cloud View: The word cloud format illustrates the prevalence of adipogenesis-related lipids, where the prominence of adipogenesis-related lipids correlates with the extent of literature evidence, with larger font sizes indicating a higher frequency of associated literature documentation.


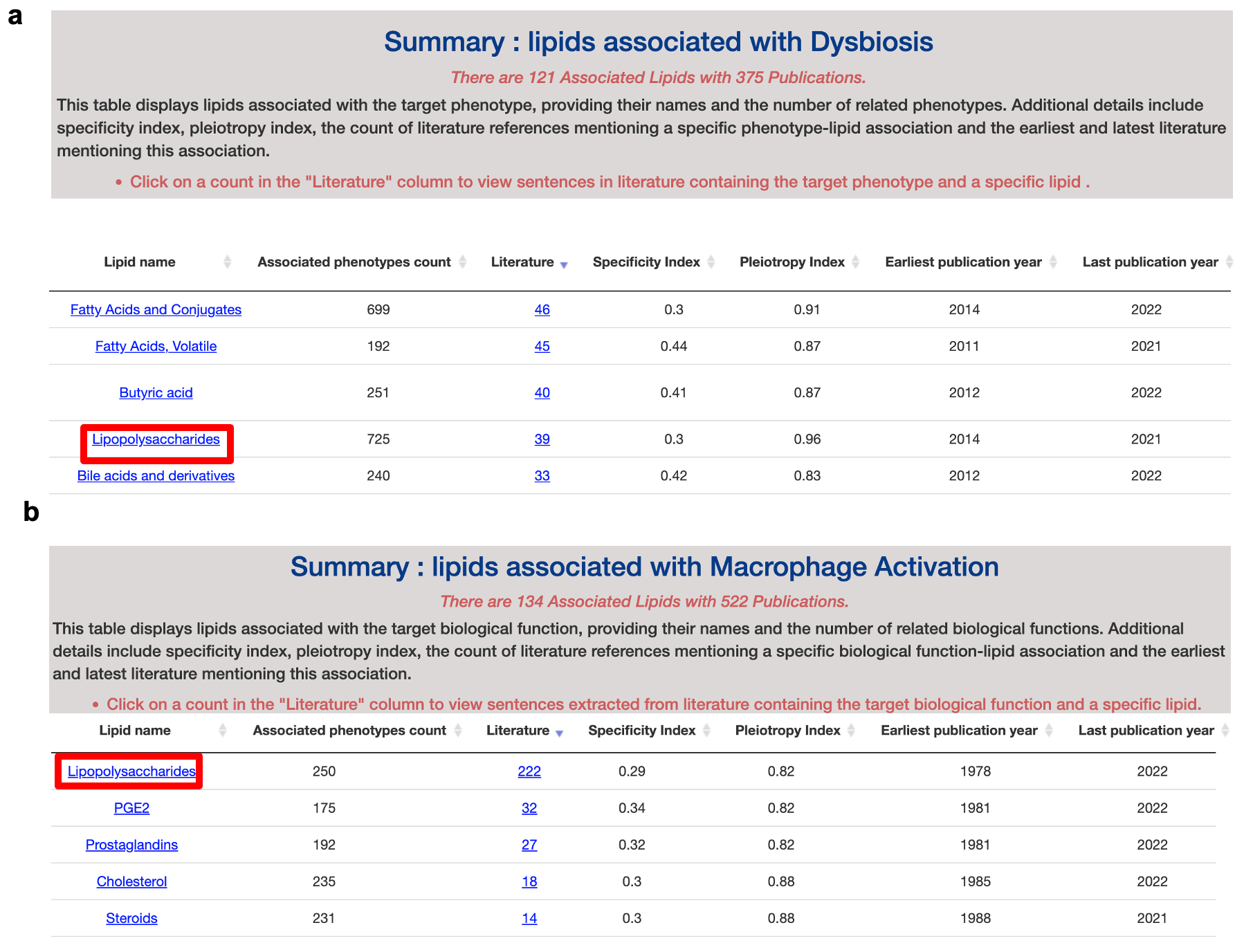


**Supplementary Figure 5.** **A demonstration of lipopolysaccharides-related phenotypes evidence in sentence-level in LipidFun.** Both overall summary of **a** dysbiosis-related lipids and **b** macrophages activation-related lipids including the number of literatures and the publication periods are depicted, respectively.

**Supplementary Figure 6**. Comparison of annotated lipids in LipidFun and LipidPedia. The figure illustrates the shared and unique lipids between the two databases, along with their respective proportions.
